# Impaired ApoB secretion triggers enhanced secretion of ApoE to maintain triglyceride homeostasis in hepatoma cells

**DOI:** 10.1016/j.jlr.2025.100795

**Published:** 2025-04-01

**Authors:** Kotomi Shinozaki, Tomoko Honda, Kenzaburo Yamaji, Emi Nishijima, Ikuyo Ichi, Daisuke Yamane

**Affiliations:** 1 Department of Diseases and Infection, Tokyo Metropolitan Institute of Medical Science, Tokyo, Japan; 2 Department of Nutrition and Food Science, Ochanomizu University, Tokyo, Japan; 3 Graduate School of Humanities and Sciences, Ochanomizu University, Tokyo, Japan

**Keywords:** ApoB, ApoE, apolipoproteins, hepatocyte, lipid transfer proteins, liver, sialylation, triglycerides, VLDL

## Abstract

Apolipoprotein B (ApoB) is essential for the assembly and secretion of triglyceride (TG)-rich VLDL particles, and its dysfunction is linked to metabolic disorders, including dyslipidemia and liver steatosis. However, less attention has been paid to whether and how other apolipoproteins play redundant or compensatory roles when the ApoB function is compromised. Here, we investigated the effects of microsomal triglyceride transfer protein (MTP), which mediates lipidation of nascent ApoB, on ApoE function. We observed a paradoxical increase in ApoE secretion resulting from increased expression in MTP inhibitor (MTPi)-treated human hepatoma cells. This phenotype was recapitulated in *APOB*-knockout cells and was associated with impaired ApoB secretion. While MTP-dependent transfer of neutral lipids is dispensable for ApoE secretion, TG biosynthesis, redundantly catalyzed by DGAT1 and DGAT2, is required for efficient ApoE secretion in hepatoma cells. ApoE colocalizes with lipid droplets near the Golgi apparatus and mediates TG export in an ApoB-independent fashion. We found that simultaneous inhibition of both ApoE and ApoB, but not inhibition of either alone, led to TG accumulation in hepatoma cells, indicating that both proteins function redundantly to control TG content. Validation studies in primary human hepatocytes (PHHs) demonstrated DGAT2-dependent secretion of ApoE. While MTPi treatment did not elevate ApoE secretion, it induced increased sialylation of ApoE in the supernatants of PHHs. These results show that enhanced ApoE secretion compensates for the impaired ApoB function to maintain the lipid homeostasis, providing an alternative route to modulate lipid turnover in hepatoma cells.

## Graphical abstract

**Figure:**
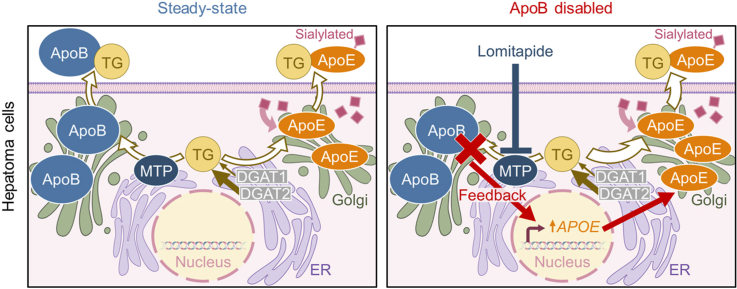

The secretion of ApoB-containing lipoproteins is initiated through the triglyceride transfer protein (MTP)-dependent co-translational lipidation of ApoB, which triggers the assembly of VLDL particles (1). The critical role of ApoB-containing lipoproteins in mediating the export of intrahepatic lipids is underscored by the strong association between genetic variations in *APOB* or *MTTP* and the development of fatty liver disease (2, 3, 4, 5, 6, 7). While the essential function of ApoB in lipid transport is well-established, the potential roles of other apolipoproteins in regulating hepatic lipid homeostasis, whether compensatory or redundant, remain less clearly defined. Recent studies have linked genetic polymorphisms in *APOE* to fatty liver disease (8, 9, 10, 11). Additionally, elevated plasma concentrations of ApoE in the HDL fraction are observed in patients with abetalipoproteinemia (12), suggesting that ApoE may play a significant role in lipid secretion in place of ApoB under certain pathological conditions. However, relatively little is known about the regulation of ApoE secretion in hepatocytes, the primary source of plasma ApoE (13).

ApoE is associated with very low-density lipoproteins (VLDL) and high-density lipoproteins (HDL) (14). Numerous reports have highlighted the crucial role of ApoE as a mark for LDLR-dependent lipoprotein uptake by peripheral tissues: deficiency of ApoE leads to dyslipidemia and atherosclerosis in mice (15, 16, 17). While relatively few studies have investigated its role in lipid export from hepatocytes, overexpression and knockout (KO) studies have demonstrated that ApoE has an intrinsic ability to stimulate the production of VLDL triglyceride (TG) in vivo and in vitro (18, 19). Previous findings have suggested that the regulatory mechanism of ApoE secretion may be cell-type specific: The majority of ApoE is sialylated in hepatocytes (20), whereas protein kinase A and calcium-dependent signals regulate ApoE secretion in macrophages (21). In astrocytes, pro-inflammatory stimuli lower ApoE secretion in an isoform-dependent manner (22). While it is likely that different post-translational modifications regulate ApoE secretion among different cell types (13), understanding the regulatory mechanism and functional roles of ApoE secretion in hepatocytes may help elucidate the mechanism underlying lipid homeostasis and the associated metabolic diseases.

This study aims to investigate the mechanisms regulating ApoE secretion and how this process contributes to hepatic TG homeostasis. We found that while ApoE can be secreted as part of ApoB-containing VLDL particles, secretion of ApoE by itself is strongly activated upon pharmacological inhibition of MTP or genetic ablation of *APOB* in human hepatoma cells. This indicates that loss of ApoB secretion triggers enhanced ApoE secretion, which occurs independently of MTP-dependent transfer of neutral lipids. However, we found both ApoB and ApoE require nascent biosynthesis of TGs catalyzed by redundant activities of both DGAT1 and DGAT2 for their efficient expression and secretion by the hepatoma cells. Our results support a model by which ApoE compensates for the loss of ApoB function through transcriptional activation and post-translational modifications and provide new insights into how hepatic lipid content is regulated by ApoE, whose abnormalities are implicated in metabolic diseases.

## Materials and Methods

### Cells

Huh-7.5 human hepatoma cells were cultured in Dulbecco’s modified Eagles’s medium (DMEM), High Glucose supplemented with 10% fetal bovine serum (FBS), 1×GlutaMAX-Ι and 1×MEM Non-Essential Amino Acids Solution (Thermo Fisher Scientific) as described previously (23). Primary human hepatocytes (PHHs) were purchased from PhoenixBio and maintained in DMEM supplemented with 10% FBS, 20 mM HEPES (Thermo Fisher Scientific), 44 mM sodium bicarbonate, antibiotics (100 U/ml penicillin G and 100 μg/ml streptomycin), 15 μg/ml L-proline (Wako), 0.25 μg/ml insulin, 50 nM dexamethasone (Sigma-Aldrich), 5 ng/ml epidermal growth factor (Millipore), 0.1 mM L-ascorbic acid (Wako), and 2% dimethyl sulfoxide (Sigma-Aldrich).

### Cell studies

For lipoprotein secretion assay, Huh-7.5 cells were seeded onto 12-well plates and treated with 2.5 μM MTPi. After incubation overnight, the medium was replaced with fresh culture medium containing MTPi or serum-free medium (DMEM supplemented with 1×GlutaMAX-Ι, 1×MEM Non-Essential Amino Acids Solution, and 1 μM α-tocopherol) containing MTPi for additional 6 h, and the culture supernatants and cell lysates harvested for immunoblot analysis. Alternatively, the culture supernatants and lysates from PHHs were harvested after incubation with the MTPi for 24 h.

### Reagents and antibodies

Primary antibodies to ApoB (1:200 dilution, sc-13538) and SOAT1 (1:200 dilution, sc-137013, non-reducing condition) were from Santa Cruz Biotechnology; ApoE (1:500 dilution, MCA5639GA) was from Bio-Rad; SOAT2 (1:1,000 dilution, 21852-1-AP, non-reducing condition), Albumin (1:50,000 dilution, 16475-1-AP), and LDLR (1:5,000 dilution, 10785-1-AP) were from Proteintech; Actin (1:10,000 dilution, A2066) and FLAG (1:1,000 dilution, F7425) were from Sigma; GAPDH (Clone 5A12; 1:5,000 dilution, 016–25523) was from Wako; IRDye 680 or 800 secondary antibodies including #926–68072, #926–32213, and #926–32212 (1:20,000 dilution) were from LI-COR. HRP-linked secondary antibody #70762P (1:5,000 dilution) was from Cell Signaling Technology. MTPi (Lomitapide, BMS-201038) and oleic acid were purchased from Cayman Chemical. GSK2033 was from Selleck. Puromycin was from Invivogen. Brefeldin A and Actinomycin D were from Wako. *O*-glycosidase and Neuraminidase (E0540S) were purchased from New England Biolabs. Cell viability was determined using Cell Counting Kit-8 (DOJINDO) on 96-well plates according to the manufacturer's protocol.

### Lentiviral plasmids, production, and transduction

To generate *APOE*/*APOB* KO Huh-7.5 cells, single guide RNA (sgRNA) sequences targeting each gene (*APOB*, 5′-AGCTGGCGATGGACCCGCCG-3′; *APOE*, 5′-GCTTTTGGGATTACCTGCGC-3′; *DGAT1* #1, 5′-CGCAGATCTTGAGCAATGCC-3′; *DGAT1* #2, 5′-AGCTCACCAATAACCAGGCA-3′; *DGAT2* #2, 5′-TCGCTGTGCTCTACTTCACT-3′; *DGAT2* #4, 5′-TCGAGACTACTTTCCCATCC-3′; *SOAT1*, 5′-TTGAGAGCACCTCCAGAACA-3′; *SOAT2*, 5′-GGTACAATGGACCCGACACA-3′) were cloned into BsmBI-digested lentiCRISPRv2 plasmid (24). The lentiviral transfer plasmids encoding *DGAT1* or *DGAT2* fused with C-terminal FLAG sequence were created by PCR amplifying the host genes using cDNA derived from Huh-7.5 cell total RNA as template and primers flanked by XbaI and NheI restriction sites as described (25). Lentivirus production was carried out by co-transfection of individual lentiviral transfer plasmids and lentiviral packaging plasmids into 293T cells. Lentiviral transduction was performed by supplementation of 8 μg/ml polybrene, followed by antibiotic selection with 6 μg/ml puromycin. Single-cell clones were isolated using limiting dilution on 96-well plates.

### RNA extraction and quantitative RT-PCR

Total RNA extraction was performed with the RNeasy mini kit (Qiagen). Quantification of *APOE*, *APOB*, *DGAT1, DGAT2, ST6GAL1,* and *ST6GALNAC6* genes was carried out by a one-step quantitative RT-PCR analysis with the Luna Universal One-Step RT-qPCR Kit (NEB) or *RNA-direct* Realtime PCR Master Mix (TOYOBO) using specific primer pairs as follows: *APOE*, forward 5′-GTCGCTTTTGGGATTACCTG-3′ and reverse 5′-TTTGTAGGCCTTCAACTCCT-3′; *APOB*, forward 5′-AGAGGACAGAGCCTTGGTGGAT-3′ and reverse 5′-CTGGACAAGGTCATACTCTGCC-3′; *DGAT1*, forward 5′-TGAGCTCATGCAGTTTGGAG-3′ and reverse 5′-CCAGTTCTGCCAGAAGTAGG-3′; *DGAT2*, forward 5′-CAAGCCCATCACCACTGTTG-3′ and reverse 5′-GGTGTGGTACAGGTCGATGT-3′; *ST6GAL1*, forward 5′-AACTCTCAGTTGGTTACCACAGA-3′ and reverse 5′-GGTGCAGCTTACGATAAGTCTT-3′; *ST6GALNAC6*, forward 5′-AGAAGTGGAGCATCACTGAC-3′ and reverse 5′-TGCATCATTCATGCGGATTG-3′.

### Immunoblots

Western blotting was performed with standard methods. Lysates were prepared in lysis buffer (50 mM Tris-HCl, pH 7.5, 150 mM NaCl, 1 mM EDTA, 1% Triton X-100, 20 mM sodium fluoride, 1 mM sodium orthovanadate) supplemented with cOmplete protease inhibitor cocktail (Roche) and resolved on a 5%–20% gradient SDS-PAGE gel (Wako). To analyze sialylation and *O*-linked glycosylation of ApoE, culture supernatants were concentrated using Amicon Ultra-0.5 ml filters (10 kDa cutoff) and were treated for 1 h at 37°C with neuraminidase and *O*-glycosidase, and then separated by SDS-PAGE. Odyssey CLx Infrared Imaging System (LI-COR Biosciences) was used for visualization and quantitation. Alternatively, LAS3000 (FUJIFILM) was also used for visualization, and the quantification of specific protein bands was performed as described previously (26). Protein expression levels were normalized to the amount of protein content, housekeeping proteins, β-actin or GAPDH, or total RNA content.

### RNA interference

siRNA pools targeting *DGAT1* (5′-UCAUGUACGUCCACGACUA-3′, 5′-UGACCUACCGCGAUCUCUA-3′, 5′-CUACCGCGAUCUCUACUAC-3′ and 5′-CGGGAGUUCUACCGGGACU-3′) and *DGAT2* (5′-UCACUUGGCUGGUGUUUGA-3′, 5′-GCAGGCAACUUCCGAAUGC-3′, 5′-GCCGAUGGGUCCAGAAGAA-3′ and 5′-GCAAUGCUAUCAUCAUCGU-3′) were obtained from Dharmacon and transfected twice (6 h and 24 h after plating) into cells using Lipofectamine RNAiMAX Transfection Reagent (Thermo Fisher Scientific) at a final concentration of 20 nM according to the manufacturer’s protocol.

### Luciferase reporter assay

The NLuc reporter vector pNL-LXRE was prepared by annealing oligonucleotides containing two tandem repeats of the LXR binding motifs derived from the *APOE* locus followed by a minimal TATA promoter, 5′-CTAGCAGGGTCACTGGCGGTCAAAGGCAGGGTCACTGGCGGTCAAAGGA-3′ and 5′-AGCTTCCTTTGACCGCCAGTGACCCTGCCTTTGACCGCCAGTGACCCTG-3′, and inserted into pNL2.3 plasmid (Promega) using NheI and HindIII restriction sites. The putative *APOE* promoter regions were amplified with PrimeSTAR GXL DNA Polymerase (TaKaRa) and the following primers (i) 5′- ACCTGAGCTCGCTAGTGCATCATACTGTTCCCAC-3′ and 5′- TATACCCTCTAAGCTGTTCCCAGCGCTGGCCGCTCTGC-3′; (ii) 5′- ACCTGAGCTCGCTAGTCACATTCCTGGCAGGTAT-3′ and 5′-TATACCCTCTAAGCTGTTCCCAGCGCTGGCCGCTCTGC-3′; (iii) 5′- ACCTGAGCTCGCTAATCCAGGAGTCCAGATCCCC-3′ and 5′- TATACCCTCTAAGCTCGGCTCCTGGGGAAGGACGTCCTT-3′ using DNA isolated from Huh-7.5 cells as template. The elements were co-transfected with NLuc reporter vector. NLuc activity was measured using Nano-Glo Luciferase Assay System (Promega) as described (27). Luminescence was analyzed on a Mithras LB940 Multimode Microplate Reader (Berthold).

### Equilibrium ultracentrifugation

Huh-7.5 cells were seeded and incubated with and without MTPi (2.5 μM) overnight. Cells were fed with fresh media containing respective compounds and incubated for 20 h. Supernatants from Huh-7.5 or *APOB*-KO2 cells were harvested and centrifuged at 1,500 *g* for 5 min and concentrated using Amicon Ultra 15 ml tube at 2,380 *g* for 15 min. Samples were then resuspended in DMEM and then layered on top of a pre-formed continuous 10%–40% iodixanol (OptiPrep, Sigma-Aldrich) gradient in Hanks’ balanced salt solution (HBSS, Gibco). Gradients were centrifuged at 35,000 rpm for 20 h at 4°C in an SW41 Ti rotor (Beckman-Coulter) for 20 h. Following ultracentrifugation, fractions (1 ml each) were collected from the top of the tube. The density of each fraction was calculated from the refractive index measured with a refractometer (ATAGO).

### Visualization of BODIPY-stained neutral lipids

Cells were washed once with PBS and fixed in 4% paraformaldehyde for 20 min at room temperature. After washing with PBS, the cells were stained for 1 h with 5 μg/ml BODIPY 493/503 (Thermo Fisher Scientific) containing 0.1 ng/ml 4′,6-diamidino-2-phenylindole (DAPI). Fluorescence intensity was quantified using BZ-X analyzer (Keyence) and normalized to the DAPI signal. Flow cytometric analysis was conducted as follows: cells were washed twice using PBS and incubated in 2 μM BODIPY 493/503 solution in PBS for 15 min at 37°C. Cells were then harvested by trypsinization, pelleted at 2,000 *g*, washed twice in PBS containing 1% FBS, and analyzed using an LSR Fortessa cytometer (BD Bioscience) and FlowJo software (FlowJo LLC).

### TG extraction and quantification

To assess TG synthesis, Huh-7.5 cells were treated with 100 μM ^13^C-OA (Cayman) and incubated overnight. The culture medium was replaced with a fresh growth medium, and 6 h later, the cell monolayer was washed with PBS and trypsinized. The cell pellets were split into two tubes for TG analysis and Bradford assay to determine protein concentrations.

The lipids were extracted from the cell pellets by the Bligh-Dyer method (28). TG was separated by thin-layer chromatography (Millipore), and fractionated TG was extracted by the Bligh-Dyer method (28) and methylated with 2.5% sulfuric acid in methanol. The methylated ^13^C-OA was analyzed by gas chromatography-mass spectrometry (GC-MS) using a QP2010 Ultra (Shimadzu). Quantification was performed using the selected ion monitoring (SIM) mode. The mass selective detector was set for SIM of m/z 266 and 297 for the methylated ^13^C-OA. The fragment ion at m/z 266 was used for quantification.

Alternatively, TG levels were quantified with the LabAssay™ Triglyceride (Wako). TG was extracted from cells plated on 6-well plates by incubating in 1 ml isopropanol overnight at 4°C. The isopropanol from each well was subsequently collected in microcentrifuge tubes, dried, and the TG was resuspended in 10–20 μl isopropanol. For quantification, 1 μl from each sample was used for the assay as per the manufacturer’s instruction. Absorbance was measured at 570 nm, and the data were normalized to protein concentrations.

### Confocal laser scanning fluorescence microscopy

Huh-7.5 cells were seeded onto an eight-well chamber slide (Watson), treated with fresh media containing DMSO or MTPi (lomitapide, 2.5 μM), and incubated overnight. Cells were then washed twice with PBS, fixed with 4% paraformaldehyde for 20 min, washed twice with PBS, and permeabilized with 0.2% Triton X-100 for 10 min. After washing with PBS, cells were blocked with Intercept Blocking Buffer for 1 h. Cells were then stained with primary antibodies to ApoE (1:100 dilution, MCA5639, Bio-Rad) and washed three times with PBS containing 0.5% Tween-20, followed by overnight incubation with Anti-PDI (1:100 dilution, ADI-SPA-890, Enzo Life Sciences) or Anti-GM130 (1:500 dilution, PM061, MBL) antibodies. After washing three times with PBS containing 0.5% Tween-20, cells were stained with secondary antibodies: Alexa Fluor 594 goat anti-mouse IgG (1:500 dilution) and Alexa Fluor 647 goat anti-rabbit IgG (1:500 dilution) for 1 h. Cell imaging was performed on TCS SP8 STED (Leica).

### Statistical analysis

Unless noted otherwise, the error bars represent the standard deviation and are shown for experiments with n = 3 or greater. All between-group comparisons were carried out by ANOVA or unpaired *t* test using Prism 10 software (GraphPad Software, Inc.). *P* < 0.05 was considered statistically significant.

## Results

### MTP inhibition promotes ApoE secretion by enhancing its synthesis

**Fig. 1:**
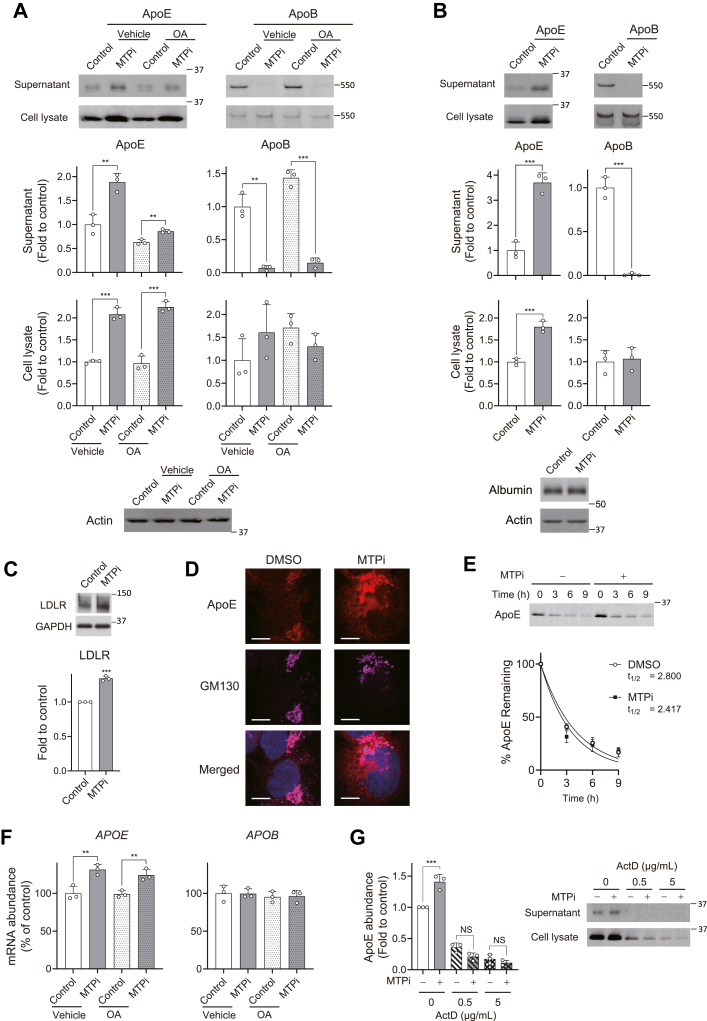
MTP inhibition promotes ApoE secretion by enhancing its expression. A: Immunoblots of ApoE and ApoB in supernatants and cell lysates of Huh-7.5 cells treated with EtOH (Vehicle) or 200 μM oleic acid (OA) in the presence or absence of 2.5 μM MTPi. Relative abundances of ApoE (left panels) and ApoB (right panels) are shown below. ∗∗*P* < 0.01, ∗∗∗*P* < 0.001 (n = 3, two-tailed Student's *t* test). B: Results of MTPi treatment as in (A) in serum-free medium. Loading controls (Actin and human albumin) are shown at the bottom. ∗∗∗*P* < 0.001 (n = 3, two-tailed Student's *t* test). C: Immunoblots of LDLR in lysates from Huh-7.5 cells treated with MTPi. ∗∗∗*P* < 0.001 versus control (n = 3, two-tailed Student's *t* test). D: Confocal microscopic images of Huh-7.5 cells treated with MTPi or DMSO control. Cells were stained with antibodies against ApoE (red) and the Golgi marker GM130 (magenta), and DAPI to stain nuclei (blue). Scale bar, 10 μm. E: Stability of ApoE protein in MTPi-treated Huh-7.5 cells after treatment with 50 μg/ml puromycin and 100 ng/ml brefeldin A. Data were fit to a one-phase decay model (n = 3, *R*^2^ = 0.9279–0.9726). F: Effect of MTPi on *APOE* and *APOB* mRNA levels in cells treated with EtOH (Vehicle) and OA. ∗∗*P* < 0.01 (n = 3, two-way ANOVA with Sidak’s multiple comparisons test). G: Inhibition of de novo transcription of *APOE* by treating with different concentrations of actinomycin D (ActD). ∗∗∗*P* < 0.001 (n = 3, two-way ANOVA with Sidak’s multiple comparisons test).

MTP is well characterized as an essential factor that transfers neutral lipids onto ApoB and promotes its secretion. In contrast, conflicting results have been reported on how MTP functions to regulate ApoE secretion (29, 30, 31, 32). To clarify the relationship between ApoE and MTP activity, we suppressed its function using the MTP inhibitor (MTPi) lomitapide in human hepatoma Huh-7.5 cells, a HuH-7 subline highly active in lipid metabolism (33), in the presence or absence of oleic acid (OA) that stimulates VLDL secretion (34). While MTPi treatment efficiently downregulated the secretion of ApoB (Fig. 1A), it induced significant enhancement of ApoE secretion (Fig. 1A). The MTPi-induced enhancement of ApoE secretion was also observed when the cells were incubated with serum-free medium (Fig. 1B). This was not due to reduced uptake of ApoE, as MTPi treatment did not reduce, but rather induced a modest increase in LDLR expression (Fig. 1C). ApoE secretion is similarly upregulated by MTPi when ApoB secretion was stimulated by OA, although the MTP-induced enhancement was lower than that observed in unstimulated cells. Increased levels of intracellular ApoE were also evident in cells treated with MTPi (Fig. 1A, B). Confocal microscopy confirmed the enhanced expression of cytosolic ApoE, with the majority localized near the Golgi marker GM130 in MTPi-treated cells (Fig. 1D). We found that MTPi treatment did not affect the half-life of ApoE protein in cells treated with puromycin and brefeldin A (Fig. 1E), while it significantly increased *APOE* mRNA levels, indicating that MTP inhibition upregulates intracellular synthesis of ApoE at least partially through transcription (Fig. 1F). Indeed, inhibition of de novo transcription by actinomycin D completely prevented increased expression of intracellular ApoE (Fig. 1G).

### Enhanced ApoE secretion induced by MTPi is phenocopied by depletion of ApoB expression

**Fig. 2:**
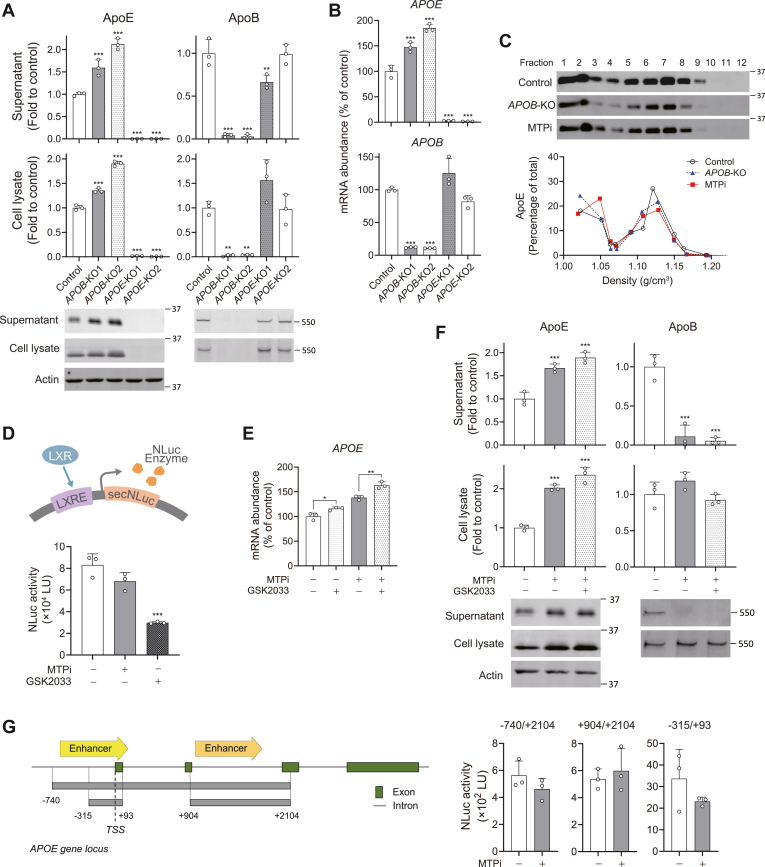
Enhanced ApoE secretion induced by MTPi is phenocopied by depletion of ApoB expression. A: Relative abundances of ApoE and ApoB in supernatants (upper panels) and lysates (lower panels) of two different *APOE*-KO and *APOB*-KO clones. Immunoblots are shown below. ∗∗*P* < 0.01, ∗∗∗*P* < 0.001 versus control (n = 3, one-way ANOVA with Dunnett’s multiple comparisons test). B: RT-qPCR determination of *APOE* and *APOB* mRNA levels in two different *APOE*- and *APOB*-KO clones. ∗∗∗*P* < 0.001 versus control (n = 3, one-way ANOVA with Dunnett’s multiple comparisons test). C: Density distribution of ApoE secreted from Huh-7.5 cells expressing control sgRNA vector, *APOB*-KO2, and control cells treated with 2.5 μM MTPi. D: Schematic representation of secretory Nanoluciferase (NLuc) reporter analysis of LXRE-NLuc transfected in Huh-7.5 cells. Effects of MTPi (2.5 μM) and GSK2033 (3 μM) on NLuc activity are shown below. NLuc activities were normalized to the cell number. ∗∗∗*P* < 0.001 versus control (n = 3, one-way ANOVA with Dunnett’s multiple comparisons test). E: RT-qPCR determination of *APOE* mRNA levels in Huh-7.5 cells treated with MTPi (2.5 μM) and GSK2033 (3 μM). ∗*P* < 0.05, ∗∗*P* < 0.01 (n = 3, two-way ANOVA with Sidak’s multiple comparisons test). F: Relative abundances of ApoE and ApoB in supernatants and lysates from Huh-7.5 cells treated with MTPi and GSK2033. Immunoblots are shown below. ∗∗∗*P* < 0.001 versus control (n = 3, one-way ANOVA with Dunnett’s multiple comparisons test). G: Schematic representation of secretory NLuc reporter constructs containing *APOE* promoter enhancer regions transfected in Huh-7.5 cells. Effects of MTPi (2.5 μM) on NLuc activity are shown. NLuc activities were normalized to the relative cell number. TSS, transcription start site. (n = 3, two-tailed Student's *t* test).

We next interrogated whether the MTPi-induced upregulation of ApoE expression is related to its inhibition of ApoB secretion. To this end, we applied CRISPR/Cas9-genome editing to disrupt genes encoding *APOB* and *APOE* and isolated multiple KO clones. Interestingly, ApoE secretion and intracellular expression levels were significantly elevated in two independent *APOB*-KO cells (Fig. 2A). *APOB*-KO cells possess significantly greater mRNA levels of *APOE* (Fig. 2B). Given that MTP inhibition and loss of ApoB expression had a similar impact on ApoE secretion, we considered that ApoE expression might be regulated by a mechanism that compensates for the loss of ApoB-dependent transport.

To examine how the presence or absence of ApoB impacts the buoyant density of ApoE, we fractionated supernatants obtained from *APOB*-KO2 and MTPi-treated cells according to the densities, and compared them with control cells. However, no notable differences in the ApoE distribution were found among the samples (Fig. 2C). Thus, the loss of ApoB expression or MTP inhibition caused quantitative changes in ApoE secretion but was not associated with significant changes in its distribution among lipoprotein fractions secreted from Huh-7.5 cells.

Several reports have shown that ApoE expression is predominantly regulated by the liver X receptor (LXR) in astrocytes, macrophages, adipocytes, and hepatoma (HepG2) cells (35, 36, 37). To determine whether MTPi activates LXR-dependent ApoE transcription, we constructed LXR reporter cells in which two tandem repeats of an LXR-responsive element (LXRE) derived from the *APOE* locus drives expression of secreted Nanoluciferase (NLuc) in response to LXR binding (Fig. 2D) (36, 38). Contrary to our expectation, MTPi caused slightly reduced LXR promoter activity, albeit at lower efficiency than the LXR antagonist GSK2033 (Fig. 2D). Furthermore, GSK2033 treatment alone slightly enhanced the transcription of *APOE*, and co-treatment with MTPi and GSK2033 did not reverse the MTPi-dependent activation of ApoE at both mRNA and protein levels (Fig. 2E, F). Collectively, these results indicate that MTPi-induced enhancement of ApoE in Huh-7.5 cells occurs independently of LXR activation. Additionally, we have also developed new reporter constructs containing *APOE* promoter enhancer regions and assessed their response to MTPi treatment (Fig. 2G). Although these constructs included regions responsive to important transcription factors, such as NFκB, c-Jun, and CEBPβ (39, 40), none of these constructs showed enhanced NLuc activity following MTPi treatment, suggesting that *APOE* transcription is regulated in a noncanonical manner, possibly involving distal regulatory elements.

### TG biosynthesis regulates the secretion of both ApoE and ApoB

**Fig. 3:**
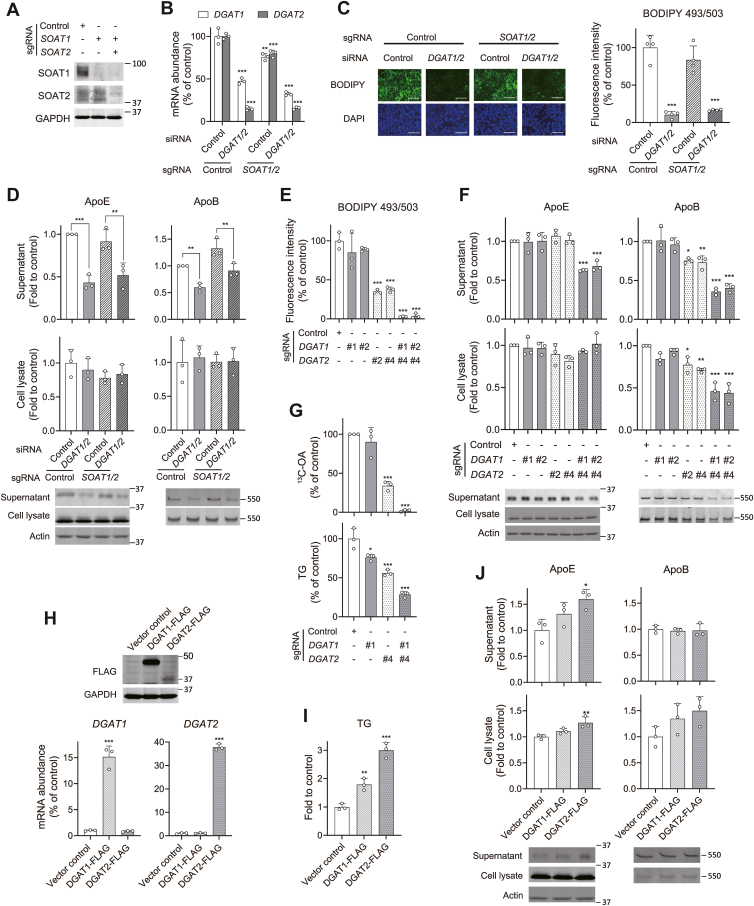
TG biosynthesis regulates the secretion of both ApoE and ApoB. A: Immunoblots showing depletion of SOAT1 and SOAT2 in Huh-7.5 cells expressing indicated sgRNAs. B: RT-qPCR determination of *DGAT1* and *DGAT2* mRNA levels in control and *SOAT1*/*2* sgRNA-expressing Huh-7.5 cells transfected with indicated siRNAs. ∗∗*P* < 0.01, ∗∗∗*P* < 0.001 versus control (n = 3, one-way ANOVA with Dunnett’s multiple comparisons test). C: BODIPY 493/503 staining of LDs (green) in Huh-7.5 cells transfected with indicated siRNAs and expressing indicated sgRNAs. BODIPY values were normalized to the cell number as measured by DAPI staining (blue). ∗∗∗*P* < 0.001 versus control (n = 4, one-way ANOVA with Dunnett’s multiple comparisons test). Scale bar, 100 μm. D: Relative abundances of ApoE and ApoB in supernatants (upper panels) and lysates (lower panels) from Huh-7.5 cells expressing indicated sgRNAs and transfected with indicated siRNAs. Immunoblots are shown below. ∗∗*P* < 0.01, ∗∗∗*P* < 0.001 (n = 3, two-way ANOVA with Sidak’s multiple comparisons test). E: Relative fluorescence intensity of Huh-7.5 cells expressing indicated sgRNAs stained with BODIPY 493/503. ∗∗∗*P* < 0.001 versus control (n = 3, one-way ANOVA with Dunnett’s multiple comparisons test). F: Relative abundances of ApoE and ApoB in supernatants (upper panels) and lysates (lower panels) from Huh-7.5 cells expressing indicated sgRNAs. Immunoblots are shown below. ∗*P* < 0.05, ∗∗*P* < 0.01, ∗∗∗*P* < 0.001 versus control (n = 3, one-way ANOVA with Dunnett’s multiple comparisons test). G: (top) Incorporation of ^13^C-OA into TG in Huh-7.5 cells expressing indicated sgRNAs. TG abundance was quantified by GC-MS. (bottom) TG abundance in the same set of cells was determined by a colorimetric assay. ∗*P* < 0.05, ∗∗∗*P* < 0.001 versus control (n = 3, one-way ANOVA with Dunnett’s multiple comparisons test). H: (top panel) Immunoblots showing expression of FLAG-tagged DGAT proteins in Huh-7.5 cells. (bottom panels) RT-qPCR determination of *DGAT1* and *DGAT2* mRNA levels. ∗∗∗*P* < 0.001 versus control (n = 3, one-way ANOVA with Dunnett’s multiple comparisons test). I: TG abundance in Huh-7.5 cells expressing DGAT1-FLAG, DGAT2-FLAG or empty vector determined by a colorimetric assay. ∗∗*P* < 0.01, ∗∗∗*P* < 0.001 versus control (n = 3, one-way ANOVA with Dunnett’s multiple comparisons test). J: Relative abundances of ApoE and ApoB in supernatants (upper panels) and lysates (lower panels) from Huh-7.5 cells expressing DGAT1-FLAG or DGAT2-FLAG. Immunoblots are shown below. ∗*P* < 0.05, ∗∗*P* < 0.01 versus control (n = 3, one-way ANOVA with Dunnett’s multiple comparisons test).

MTP mediates the transfer of neutral lipids, TG and cholesteryl ester (CE), to the core of nascent ApoB, thereby promoting its secretion (41). Given the fact that MTPi increased ApoE secretion (Fig. 1A, B), we hypothesized that the ApoE secretory process might be regulated independently of MTP-mediated transfer of neutral lipids. To determine whether the biosynthesis of TG or CE affects ApoE expression and secretion, we depleted the expression of diacylglycerol acyltransferase (DGAT) and sterol O-acyltransferase (SOAT) enzymes that catalyze the final step of TG and CE synthesis, respectively. DGAT1 and DGAT2, two isoforms of DGAT, were simultaneously silenced by transfecting with specific siRNA pools in control Huh-7.5 cells or in *SOAT1*/*SOAT2*-double KO (DKO) cells generated by CRISPR/Cas9 genome editing (Fig. 3A, B). Efficient knockdown of both *DGAT* genes and a concomitant reduction in TG levels were validated (Fig. 3B, C). Silencing *DGAT* genes caused significantly reduced secretion of ApoB and ApoE without affecting intracellular levels in both control and *SOAT1*/*SOAT2*-DKO cells (Fig. 3D). Together, these results show that TG biosynthesis is dominant over that of CE in promoting secretion of both ApoB and ApoE, and that newly synthesized TG enhances ApoE secretion.

To determine whether the TG biosynthesis that promotes the lipoprotein secretion is catalyzed by either DGAT1 or DGAT2, or by redundant activities of both, we examined ApoB and ApoE secretion from cells transduced with multiple sgRNAs targeting either *DGAT1* or *DGAT2*, or two sgRNAs targeting both genes. Since commercial antibodies tested were unable to detect endogenous DGAT1 and DGAT2, KO efficiency was functionally validated with quantification of intracellular TG by staining with BODIPY 493/503 (Fig. 3E). While KO of DGAT2, but not DGAT1, partially reduced TG levels, depletion of both DGAT1 and DGAT2 resulted in near-complete ablation of cellular TG levels (Fig. 3E). This was validated in two independent cell lines expressing different combinations of *DGAT1* and/or *DGAT2* sgRNAs. Consistent with the critical role of TG in promoting ApoB secretion, depletion of DGAT2, but not DGAT1, reduced ApoB secretion by approximately 25% (Fig. 3F). In contrast, simultaneous depletion of both DGAT1 and DGAT2 caused a more dramatic reduction in ApoB secretion (∼40%) and intracellular abundance (∼50%). The steady-state level of ApoE secretion, however, remained unchanged in DGAT2-depleted cells and was significantly affected only when the intracellular TG pool was nearly completely depleted by the simultaneous depletion of both DGAT1 and DGAT2 (Fig. 3F).

To precisely assess the effect of de novo TG synthesis on the secretion of ApoE and ApoB, we used isotope-labeled oleic acid (^13^C-OA) to measure TG synthesis in DGAT-depleted cells. Cells were incubated with ^13^C-OA, and the incorporation of ^13^C-OA into TG was quantified by GC-MS (Fig. 3G). The incorporation of ^13^C-OA was significantly reduced by 60% in DGAT2-depleted cells and was nearly abolished in *DGAT1*/*DGAT2*-DKO cells. The colorimetric assay similarly confirmed impaired TG synthesis in *DGAT1*/*DGAT2*-DKO cells (Fig. 3G).

To examine the effects of increased TG synthesis on ApoE and ApoB secretion, we ectopically expressed DGAT1 and DGAT2 tagged with C-terminal FLAG epitope (DGAT1-FLAG, DGAT2-FLAG) (Fig. 3H). We confirmed the expression of the FLAG-tagged proteins using FLAG antibody and found that DGAT2 has a superior function in increasing TG synthesis over DGAT1 (Fig. 3I), consistent with the results of KO experiments (Fig. 3E, G). In support of the functional importance of TG synthesis on ApoE secretion, increased TG synthesis led to enhanced accumulation of ApoE in the supernatant (Fig. 3J). In contrast to ApoE, ApoB secretion remained unaffected by the overexpression of either DGAT1 or DGAT2, and its intracellular abundance was only slightly increased (Fig. 3J). These results suggest that the steady-state levels of DGATs are sufficient to fully stimulate the ApoB secretion, and increasing the DGAT1 or DGAT2 expression 15-fold or 35-fold, respectively (Fig. 3H), over endogenous levels did not further stimulate it.

### ApoB and ApoE redundantly contribute to TG turnover

**Fig. 4:**
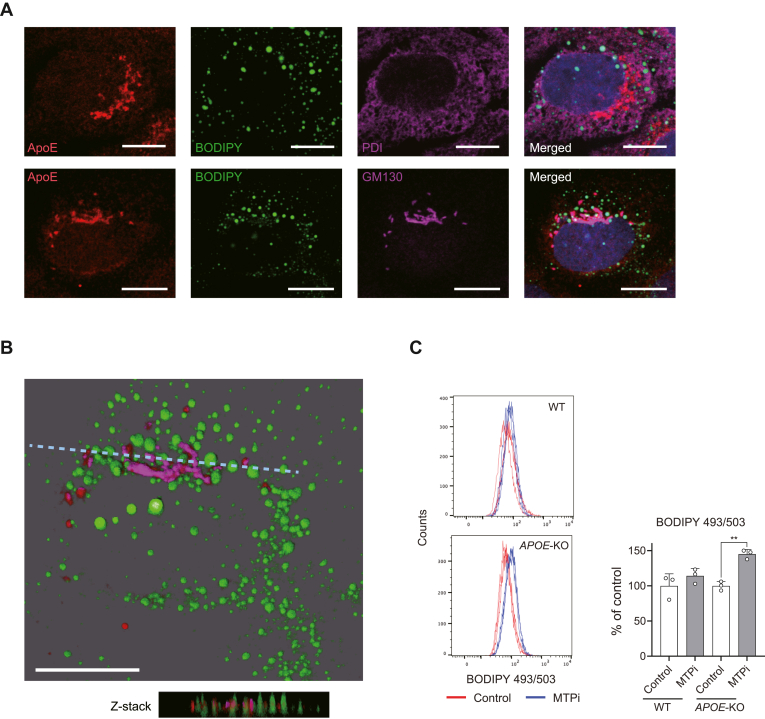
ApoB and ApoE redundantly contribute to TG turnover. A: Subcellular localization of ApoE and TG in Huh-7.5 cells. Cells were stained with antibodies against ApoE (red), the ER marker PDI (magenta), and the Golgi marker GM130 (magenta). TG and nuclei were stained with BODIPY 493/503 (green) and DAPI (blue), respectively. Scale bar, 10 μm. B: Three-dimensional (3D) view of a merged image shown in A (lower panel). Dashed lines indicate the position of the Z-stack images shown below. Scale bar, 10 μm. C: Flow cytometric analysis of BODIPY 493/503 staining in control or *APOE*-KO1 Huh-7.5 cells treated with MTPi (2.5 μM) or DMSO. ∗∗*P* < 0.01 (n = 3, two-way ANOVA with Sidak’s multiple comparisons test).

We next employed high-resolution confocal microscopy to identify the subcellular localization of ApoE and TG. While results shown in Fig. 1D and previous reports have shown that ApoE is predominantly localized to the Golgi (42, 43), others indicated that ApoE is also found in the ER lumen (44). We confirmed that ApoE is concentrated on the Golgi apparatus and partially colocalized with the ER luminal protein disulfide isomerase (PDI) (Fig. 4A). Furthermore, three-dimensional (3D) reconstruction of Z-stack images showed that BODIPY-stained TG signals were found in close proximity to the Golgi-localized ApoE (Fig. 4B). These results support the idea that TG-loaded ApoE is transported to the extracellular milieu through the Golgi apparatus.

We next determined whether and how ApoE-mediated export of TG, in parallel with TG secretion by the ApoB-dependent pathway, functions to prevent excessive accumulation of intracellular TG levels. Consistent with a predominant role for MTP-dependent secretion of ApoB-containing lipoproteins in secreting hepatic TG (45, 46), depletion of ApoE alone did not elevate intracellular TG levels as quantified by flow cytometry analysis using BODIPY dye (Fig. 4C). However, somewhat surprisingly, intracellular TG accumulation was not increased by MTPi alone in normal Huh-7.5 cells, suggesting that ApoB dysfunction is not sufficient by itself to block TG secretion. In contrast, MTPi treatment in *APOE*-KO cells significantly elevated TG accumulation (Fig. 4C). Collectively, these findings suggest that the enhanced ApoE secretion in MTPi-treated cells provides a compensatory mechanism for the ApoB-mediated TG release dysfunction and that ApoE and ApoB contribute redundantly to prevent excessive accumulation of TG in Huh-7.5 cells.

### Effects of MTPi and DGAT depletion on ApoE secretion in primary human hepatocytes

**Fig. 5:**
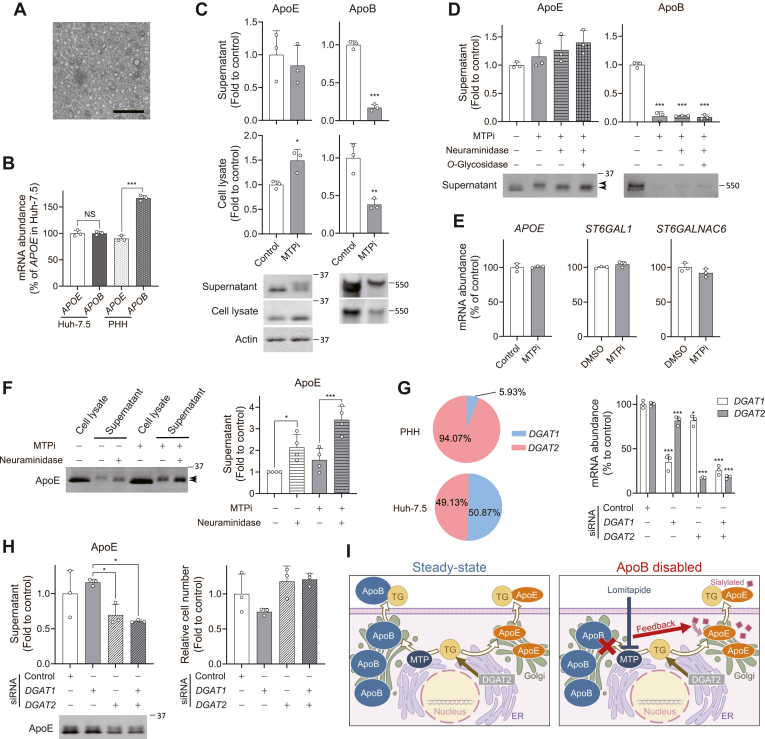
Effects of MTPi and *DGAT1/2* depletion on ApoE secretion in PHHs. A: Phase contrast microscopy of primary human hepatocytes (PHHs). Scale bar, 50 μm. B: RT-qPCR determination of *APOE* and *APOB* mRNA levels in Huh-7.5 cells versus PHHs. ∗∗∗*P* < 0.001 (n = 3, two-way ANOVA with Sidak’s multiple comparisons test). C: Relative abundances of ApoE and ApoB in supernatants and lysates of PHHs treated with MTPi (2.5 μM) or DMSO. Immunoblots are shown below. ∗*P* < 0.05, ∗∗*P* < 0.01, ∗∗∗*P* < 0.001 versus control (n = 3, two-tailed Student's *t* test). D: Effects of neuraminidase and *O*-glycosidase treatments on the post-translational modification of ApoE. Relative abundances of ApoE and ApoB in supernatants of PHHs treated as indicated. Immunoblots are shown below. Upper and lower arrowheads show sialylated and desialylated ApoE. ∗∗∗*P* < 0.001 versus control (n = 3, one-way ANOVA with Dunnett’s multiple comparisons test). E: RT-qPCR determination of *APOE*, *ST6GAL1*, and *ST6GALNAC6* mRNA levels in PHHs treated with MTPi. (n = 3, two-tailed Student's *t* test). F: Immunoblots of ApoE in supernatants and lysates from Huh-7.5 cells treated with neuraminidase as indicated. Upper and lower arrowheads show sialylated and desialylated ApoE. Relative abundance of ApoE is shown on right. ∗*P* < 0.05, ∗∗∗*P* < 0.001 (n = 4, two-way ANOVA with Sidak’s multiple comparisons test). G: Relative abundances of *DGAT1* and *DGAT2* mRNAs in Huh-7.5 cells versus PHHs (left). *DGAT1* and *DGAT2* mRNA levels in PHHs transfected with indicated siRNAs are shown on right. ∗*P* < 0.05, ∗∗∗*P* < 0.001 versus control (n = 3, one-way ANOVA with Dunnett’s multiple comparisons test). H: Relative abundance of ApoE in supernatants of PHHs transfected with indicated siRNAs (left panel). Immunoblots are shown below. ∗*P* < 0.05 (n = 3, one-way ANOVA with Dunnett’s multiple comparisons test). Relative cell numbers of PHHs transfected with indicated siRNAs as estimated by total RNA content are shown on right. I: Scheme showing the sialylation of secreted ApoE in response to disabled ApoB secretion in PHHs.

Finally, we aimed to validate our findings using relevant primary human hepatocytes (PHHs) (Fig. 5A). We confirmed that both PHHs and Huh-7.5 cells share the same *APOE* genotypic background (*APOE3/APOE3*). PHHs had a significantly higher level of *APOB* than *APOE* transcripts, whereas Huh-7.5 cells were found to express both transcripts at similar levels (Fig. 5B). Despite the differential expression of ApoB versus ApoE in PHHs, inhibiting ApoB secretion by MTPi caused intracellular accumulation of ApoE (Fig. 5C). However, MTPi-treatment in PHHs did not cause elevated secretion of ApoE, but instead induced slowly migrating bands of extracellular ApoE that underwent post-translational modification that enables MTP- and ApoB-independent secretion (Fig. 5C). We found that the slowly migrating bands likely represent a sialylated form of ApoE, as they were sensitive to neuraminidase treatment (Fig. 5D). MTPi-treated cells did not exhibit increased expression of *APOE* or *ST6GAL1* and *ST6GALNAC6*, key sialyltransferases expressed in the liver (47, 48), indicating post-transcriptional control of ApoE secretion (Fig. 5E). Whereas the sialylation event was observed only after treatment with MTPi in PHHs, we noticed that Huh-7.5 cells constitutively secrete slowly migrating ApoE bands even without MTPi treatment (Fig. 5F). Importantly, neuraminidase treatment converted the higher molecular weight form of ApoE to a faster migrating band, which indicates that ApoE secreted from Huh-7.5 cells is constitutively modified by sialylation. We also noticed that recognition of ApoE by the monoclonal antibody (WUE-4) was significantly improved after neuraminidase treatment, suggesting that the amino acid residues within the epitope region (140-160aa of ApoE) may be modified by sialylation, thereby decreasing the binding affinity of the antibody (Fig. 5F). These results highlight the differential post-translational modification of ApoE between PHHs and Huh-7.5 cells.

It is well established that DGAT2 plays a dominant role in TG synthesis in the liver (49, 50). Indeed, RT-qPCR determination of both transcripts revealed that *DGAT2* mRNA accounts for approximately 95% of total *DGAT* genes expressed in PHHs (Fig. 5G). This is in stark contrast with Huh-7.5 cells that express *DGAT1* and *DGAT2* mRNAs at similar levels. Consistent with these observations, we found that siRNA-mediated depletion of *DGAT2* alone, but not *DGAT1*, caused reduced secretion of ApoE in PHHs, and additional depletion of *DGAT1* did not further reduce it (Fig. 5G, H). These results highlight the differential contributions of the two DGATs as well as the MTPi-induced regulation of post-translational modifications of ApoE in hepatoma (Huh-7.5) cells versus PHHs (Fig. 5I).

## Discussion

While ApoB is essential for initiating lipid recruitment into VLDL particles, our results highlight an important compensatory role for ApoE in preventing excessive TG accumulation in response to defective ApoB secretion in hepatoma cells. We found that when ApoB secretion is impaired by MTPi or genetic depletion, the cells trigger the ApoE activation program that compensates for the compromised lipid transport. This compensatory response to ApoB dysfunction activates ApoE expression, at least partially through the upregulation of transcription in hepatoma cells. However, the molecular sensor that activates *APOE* transcription in response to MTP inhibition remains unidentified and requires further investigation.

We demonstrated that MTP-independent ApoE secretion occurs in both human hepatoma cells and physiologically relevant PHHs. Notably, MTPi treatment in PHHs induced a post-translational modification, particularly sialylation, of secreted ApoE. The sialylated form of ApoE exists in an isoform-dependent manner, with the order E2>E3>E4 (51, 52), whereas the opposite trend (E2<E3<E4) has been observed for the binding affinity to LDLR (53). This inverse relationship between ApoE sialylation and LDLR affinity suggests that increased sialylation may reduce ApoE uptake by the LDLR, thereby preventing TG accumulation in hepatocytes (51, 52, 53). In fact, studies have shown that increased sialylation of ApoE correlates with elevated plasma ApoE and TG levels in humans with acquired immunodeficiency syndrome (54). Furthermore, liver-specific depletion of ST6GAL1 has been associated with fatty liver disease (48), highlighting sialylation as a key regulator of lipid turnover in hepatocytes. While extensive sialylation was observed for secreted but not cell-associated ApoE in MTPi-treated PHHs, ApoE secreted from Huh-7.5 cells was constitutively sialylated, even in the absence of MTP inhibition (Fig. 5F). This enhanced sialylation of ApoE may reflect compromised function of ApoB in Huh-7.5 cells, due to the relatively low expression levels of *APOB* and poor lipidation compared to ApoB in PHHs (55, 56). Taken together, our results suggest that the ApoE secretory pathway is functionally activated in response to the loss of ApoB secretion.

The genetic abnormalities in *APOB* and *MTP* are well-established to be associated with the development of familial hypobetalipoproteinemia (FHBL) and abetalipoproteinemia (ABL), respectively (57), both of which are characterized by impaired ApoB secretion. Consistent with our findings in vitro (Fig. 2A), increased plasma levels of ApoE have been observed in ABL patients (12). Interestingly, other studies have shown that FHBL pathology can also be influenced by the *APOE* genotype: Individuals with at least one *APOE2* allele are at increased risk for developing FHBL (58). This finding aligns with a recent report indicating that ApoE2 has a lower capacity to transport TG compared to ApoE3 or ApoE4 (37) and patients with rs429358-T genotypes for *APOE2*/*E3* show a high tendency to have fat accumulation in the liver (11), underscoring the clinical relevance of ApoE in modulating hepatic TG levels. Furthermore, recent genome-wide association studies involving human subjects consistently identified the genetic polymorphism rs429358 in *APOE*, which encodes the missense mutation p.Cys112Arg in APOE (the *APOE4* allele). This variant has been linked to lower fat content (protective) in the liver (8, 9, 10). These findings strongly support the idea that the ability of ApoE to bind and transport TG influences susceptibility to fatty liver disease. Indeed, malfunction of ApoE has been associated with the onset of MASLD, characterized by abnormal TG accumulation in the liver in mouse models, particularly those fed a Western diet (59, 60, 61, 62).

The *APOE* genotype is also implicated in the development of diseases in other tissues. The *APOE3* genotype is most prevalent (>75%) among the human population (63), whereas the *APOE4* genotype is linked to an increased risk of neurological disease, characterized by increased TG accumulation in microglia (64). Among the ApoE isoforms, ApoE4 has an exceptionally high affinity for TG and can transport TG via its N-terminal helical domains in astrocytes (37). Therefore, understanding the regulatory mechanisms of TG secretion, particularly those involving an ApoE-dependent pathway as an independent factor, could provide important insights into the pathogenesis of diseases associated with abnormal TG metabolism.

While MTP-mediated transfer of TG onto ApoB is well-established as a critical step in VLDL assembly, the specific roles of DGAT1 and DGAT2 in regulating ApoE-containing lipoproteins remain unclear. In contrast to the well-characterized functions of DGAT1 in initiating TG synthesis in the ER and DGAT2 in expanding cytosolic TG within lipid droplets (65), we found that both DGAT1 and DGAT2 function redundantly in promoting ApoE secretion in Huh-7.5 cells. Consistent with this, simultaneous knockdown of both DGAT1 and DGAT2, but not either alone, reduced ApoE secretion (Fig. 3F). Indeed, we found that ApoE is colocalized with TGs near the Golgi apparatus (Fig. 4B). In contrast to Huh-7.5 cells, where both DGAT1 and DGAT2 contribute to DGAT activity, DGAT2 accounts for the majority of DGAT activity in PHHs, as in the liver (50, 66). Thus, silencing DGAT2 expression alone caused reduced ApoE secretion (Fig. 5H). These results underscore the differential expression status of *DGAT1* and *DGAT2* in human hepatoma cells versus PHHs, highlighting the relevance of DGAT2 in TG metabolism in the liver.

Lomitapide, a selective MTPi used in this study, was approved in 2012 for the treatment of Homozygous Familial Hypercholesterolemia (HoFH) (67). While lomitapide effectively reduces serum cholesterol and TG levels in most patients, liver toxicity due to fat accumulation occurs in only 17% of patients (68), suggesting the existence of an alternative TG secretion pathway. This is further supported by an independent study, which found that lomitapide does not frequently induce severe steatosis (69). These observations highlight the potential importance of investigating whether ApoE-dependent lipid secretion plays a crucial role in the therapeutic effects of lomitapide in HoFH patients.

In summary, our results presented in this study reveal the existence of an intrinsic feedback mechanism that, in response to ApoB dysfunction, regulates the synthesis and post-translational modification of ApoE to maintain hepatic lipid homeostasis. Furthermore, we uncover an unexpected role for the ApoE-dependent secretion pathway as a compensatory mechanism that bypasses defects in ApoB function. Of particular interest is the potential exploitation of this secretory mechanism by a hepatotropic virus, whose infectious particle maturation is redundantly assembled by ApoE and ApoB (29, 31, 32). Overall, our findings provide important insights into the molecular mechanisms underlying the pathogenesis of lipid secretion defects and the genetic abnormalities associated with ApoE function.

## Data availability

All data supporting this study are included in the manuscript.

## Conflict of interest

The authors declare that they have no conflicts of interest with the contents of this article.
